# Surgical Disparities of Parathyroid Carcinoma: Long-Term Outcomes and Deep Excavation Based on a Large Database

**DOI:** 10.1155/2021/8898926

**Published:** 2021-05-27

**Authors:** Ling Zhou, Yihui Huang, Wen Zeng, Sichao Chen, Wei Zhou, Min Wang, Wei Wei, Chao Zhang, Jianglong Huang, Zeming Liu, Liang Guo

**Affiliations:** ^1^Department of Plastic Surgery, Zhongnan Hospital of Wuhan University, Wuhan 430071, China; ^2^Department of Ophthalmology, Zhongnan Hospital of Wuhan University, Wuhan 430071, China; ^3^Department of Pediatrics, St. John Hospital and Medical Center, Detroit, MI 48236, USA; ^4^Department of Cardiovascular Surgery, Union Hospital, Tongji Medical College, Huazhong University of Science and Technology, Wuhan 430030, China

## Abstract

**Purpose:**

Parathyroid carcinoma (PC) is an uncommon endocrine disease, and surgery is considered the only potential cure. PC does not have a mature staging system because of the small number of PC patients. Our aim is to further investigate the prognostic factors associated with PC and explore the optimal extent of resection for PC patients.

**Methods:**

Univariate and multivariate Cox regression analyses were conducted to explore the influence of relevant factors on cancer-specific survival (CSS) and overall survival (OS). Survival curves were generated using the Kaplan–Meier method and analyzed using the log-rank test. The mortality rates per 1,000 person-years were calculated to evaluate patients' follow-up data. We also performed subgroup analysis based on the extent of resection.

**Results:**

The extent of resection was related to both CSS and OS, whereas race and extent of disease had a significant positive correlation with OS (all *P* < 0.05). Patients who underwent parathyroidectomy had remarkably better CSS and OS than patients who did not undergo definitive treatment.

**Conclusion:**

The extent of resection is related to CSS and OS in patients with PC. No significant difference in prognosis was observed between patients who underwent parathyroidectomy and those who underwent en bloc resection, which may provide useful parameters for the treatment of PC.

## 1. Introduction

Parathyroid carcinoma (PC) is an uncommon endocrine disease, accounting for less than 1% of primary hyperparathyroidism cases and 0.005% of all cancers [1–5]. The incidence of PC rose from 2 cases per 10 million in 1973 to 11 cases per 10 million in 2001. However, since 2001, the incidence has remained stable, between 10 and 13 cases per 10 million [6].

This rare tumor usually occurs during the fifth decade of life and presents with signs and symptoms of hyperparathyroidism, including digestive complications and renal and skeletal involvement [3, 5, 7–9]. The course of PC tends to be indolent but progressive [10]. Patients with PC usually present with serum calcium greater than 14 mg/dL, which is higher than that in patients with benign parathyroid tumors and a parathyroid hormone (PTH) level at least twice the upper limit of normal [11]. There are no preoperative markers for PC, and surgery is considered the only potential cure [4, 12–15]. Radical surgery with microscopically negative margins (en bloc resection) is recommended [16]. The extent of en bloc resection should include not only the tumor but also the ipsilateral thyroid lobe with gross clear margins and the adjacent involved structures. The tracheoesophageal, paratracheal, and upper mediastinal lymph nodes should be excised [4]. However, previous studies have revealed that the extent of thyroid resection did not affect mortality [2, 14].

Unlike other types of carcinoma, PC does not have a mature staging system, in large part because there are so few PC patients. Previous studies have reported that the overall survival (OS) of PC patients is associated with age at diagnosis, male sex, positive nodal status, and primary tumor size, and cancer-specific survival (CSS) is related to tumor size and extent of disease [2, 16–18]. However, there is much uncertainty regarding which factors should be selected in a staging system, and there are few studies assessing the relationship between the extent of resection and CSS or OS in a large sample. To further investigate the prognostic factors associated with PC and explore the optimal extent of resection, we performed a retrospective cohort analysis with 593 patients, one of the largest cohorts to date.

## 2. Materials and Methods

This retrospective study used data from the National Cancer Institute's Surveillance, Epidemiology, and End Results (SEER) Program. Since SEER is a publicly available database with anonymized data, no ethical review was required. The SEER database collects age, gender, race, primary tumor site, grade, lymph node (LN) status, extent of disease, treatment, OS, disease-specific survival, and survival months.

Our study included patients diagnosed with PC from 1973 to 2015. Using the ICD-10-CM diagnosis code C75.0 (malignant neoplasm of the parathyroid gland) for selection, 593 patients with PC were identified. Patients whose primary tumor was not PC were excluded. Based on the codes offered by SEER, the demographic parameters were defined as sex (male and female), race (white, black, and other or unknown), age at diagnosis (segmented into five groups), and year of diagnosis (divided into two groups). Pathologic parameters included primary tumor site, LN status, and extent of disease. Treatment characteristics were defined based on whether patients underwent surgery or radiation, and the extent of surgical resection was further divided into no definitive treatment, parathyroidectomy, en bloc radical resection, and debulking, according to the SEER code.

CSS and OS were the primary outcomes. Univariate Cox regression analyses were performed to examine whether CSS and OS were associated with age, gender, race, year of diagnosis, primary tumor site, grade, LN status, extent of disease, radiation treatment, or type of surgery. Based on the results of univariate Cox regression analyses, we further conducted multivariate Cox regression analyses to explore the concurrent influence of relevant factors on CSS and OS. Survival curves were generated using the Kaplan–Meier method and analyzed using the log-rank test. The cancer-specific mortality and all-cause mortality rates per 1,000 person-years were calculated to evaluate patients' follow-up data. We further performed subgroup analysis based on the extent of resection using univariate and multivariate Cox regression analysis and Kaplan–Meier analysis to generate survival curves. All the statistical analyses were performed using SPSS software (version 23.0), StataSE software, and GraphPad Prism software (version 6). A two-tailed *P* value <0.05 was considered significant.

## 3. Results

### 3.1. Demographic and Clinicopathological Characteristics

Five hundred and ninety-three patients with PC identified in the SEER registry from 1973 to 2015 were included in this retrospective cohort. Most patients were under 60 years old, although 37.6% of patients were between 45 and 59 years old. Men (*n* = 299, 50.4%) and women (*n* = 295, 49.6%) accounted for nearly the same proportion. The majority of patients were white (*n* = 499, 75.7%). Most patients (*n* = 553, 93.3%) underwent surgery, whereas a few patients (*n* = 57, 9.6%) underwent radiation therapy. Parathyroidectomy (*n* = 384, 64.8%) was the most common type of surgical resection, followed by en bloc radical resection (*n* = 38, 6.4%) and debulking (*n* = 5, 0.8%). The medium survival was 83 (range 34–146.75) months. The cancer-specific mortality rate was 9.6%, and the overall mortality rate was 35.1% (Table 1).

### 3.2. Clinicopathological Parameters That Affected CSS and OS

The univariate Cox regression analyses revealed that both CSS and OS were associated with age and extent of resection (all *P* < 0.05, Table 2). On multivariate Cox regression analysis, year of diagnosis and extent of resection were related to CSS, whereas race, extent of disease, and extent of resection were significant predictors of OS (all *P* < 0.05, Table 3). Metastatic disease was also a significant predictor of OS (univariate HR: 6.604, 95% CI: 2.296–18.999, *P* < 0.001; multivariate HR: 6.954, 95% CI: 2.163–22.355, *P*=0.001; Tables 2 and 3). However, age was not significantly correlated with CSS or OS after adjustment in multivariate Cox regression analyses (all relevant *P* > 0.05, Table 3).

Patients who underwent parathyroidectomy had remarkably better CSS (univariate HR: 0.083, 95% CI: 0.039–0.177, *P* < 0.001; multivariate HR: 0.053, 95% CI: 0.009–0.297, *P*=0.001; Tables 2 and 3) and OS (univariate HR: 0.299, 95% CI: 0.175–0.511, *P* < 0.001; multivariate HR: 0.194, 95% CI: 0.056–0.677, *P*=0.001; Tables 2 and 3) than patients who did not undergo definitive treatment.

### 3.3. Cancer-Specific and All-Cause Mortality Rates

We further generated the cancer-specific mortality and all-cause mortality rates per 1,000 person-years and the survival curves based on the significant factors in multivariate Cox regression analyses. The cancer-specific mortality rates per 1,000 person-years of patients who underwent parathyroidectomy (6.670, 95% CI: 4.146–10.729) and en bloc radical resection (7.648, 95% CI: 1.913–30.581) were similar, whereas the cancer-specific mortality rates per 1,000 person-years of patients who underwent no definitive treatment (45.504, 95% CI: 21.693–95.449) or debulking (52.863, 95% CI: 7.447–375.281) were fairly high (Table 4). As shown in Table 4, the all-cause mortality rate per 1,000 person-years of patients who underwent no definitive treatment (73.544, 95% CI: 33.041–163.701) was nearly twice as high as those of patients who underwent parathyroidectomy (34.524, 95% CI: 28.015–42.547) or en bloc radical resection (38.241, 95% CI: 20.576–71.073).

### 3.4. Subgroup Analysis of Extent of Resection

**Figure 1:**
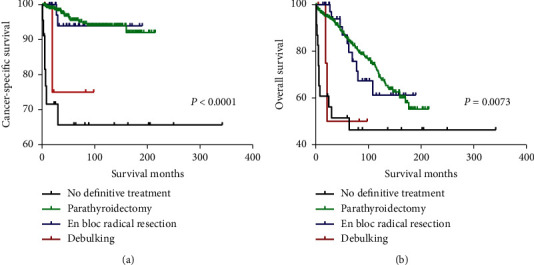
Kaplan–Meier curves among patients stratified by a different extent of resection for cancer-specific survival (a) and overall survival (b).

Survival curves for CSS and OS based on the extent of resection are shown in Figure 1. There was a significant difference in both CSS and OS based on the extent of resection (all *P* < 0.05, Figure 1). In order to further analyze the relationship of different surgeries with survival, we conducted a subgroup analysis using univariate and multivariate Cox regression analysis using different surgical methods as references and generated survival curves. Supplemental Figures 1–3 present the survival curves of patients who underwent the three different surgeries. Patients who underwent parathyroidectomy and en bloc resection had better survival than those who did not undergo definitive treatment.

Tables 5 and 6 show the results of subgroup univariate and multivariate Cox regression analysis based on the extent of resection. There was no significant difference in prognosis between patients undergoing parathyroidectomy and en bloc resection in either the survival curves or the Cox regression analysis (Tables 5 and 6 and Supplementary Figure 2). Compared to patients who underwent parathyroidectomy or en bloc resection, the prognosis of patients who underwent debulking seems not remarkably altered (Supplemental Tables 1 and 2, Supplemental Figure 3).

## 4. Discussion

In this study, we retrospectively reviewed the course of 593 PC patients. Harari et al. observed that men were more likely to develop PC [14]. However, most studies have found that the gender distribution is nearly equal [2, 6, 19, 20], as did our study. Lee et al. observed that female gender was significantly associated with an improved OS rate [2], whereas no correlation between gender and survival was found in this study. We believe that this can be explained by the difference in sample size.

Most studies examining PC are case reports; retrospective cohort studies account for only a small proportion. Several retrospective cohort studies investigating the prognosis of PC have been performed, but all had a relatively small number of subjects [2, 12, 14, 21]. In this study, 593 patients were included, and we focused on not only CSS but also OS, allowing us to comprehensively consider survival. With a large sample capacity and a primary outcome of CSS and OS, we were able to acquire a better understanding of PC.

Data from several sources have identified that the survival of patients with PC is associated with distant metastasis [2, 6, 14]. According to Lo et al., tumor size is related to CSS, whereas the opposite result was found by Harari et al. [6, 14]. With the purpose of contributing to the staging system for PC, we observed that distant metastasis was associated with worse OS in both univariate and multivariate Cox regression analysis. However, we failed to detect a connection between distant metastasis and CSS. We supposed that the main reason why distant metastasis severed as a prognosis factor for OS but not CSS is that patients with distant metastasis temp to complicate with other organ diseases and function failure. These patients may die of other organ diseases rather than parathyroid carcinoma itself. Furthermore, race was an independent risk factor for OS. Black people and patients with metastatic disease appeared to have poorer OS. This may offer a reference for choosing the factors included in the staging system of PC, but stronger proof derived from larger samples is needed.

We also extensively examined the relationship between the extent of surgical resection and survival. Surgical resection is the only curative treatment for patients with PC. Simple tumor excision (parathyroidectomy) and en bloc resection are the two most common surgical types [2]. Previous research by Harari et al. showed that the extent of thyroid resection was not associated with mortality [14]. In this study, patients who underwent parathyroidectomy and those who underwent en bloc resection had better survival than patients without definitive treatment. In addition, we also found that there was no significant difference in prognosis between patients who underwent parathyroidectomy and those who underwent en bloc resection, which is in accordance with the results of previous studies [2, 14, 21]. Consequently, we also doubt that en bloc resection is a better choice than parathyroidectomy. The resection of the ipsilateral thyroid lobe used to be recommended, but it became controversial after the report that removing the adjacent thyroid lobe in itself was not associated with improved survival [16]. In addition, it has been suggested that radical lymph node dissection is not recommended due to the low incidence of cervical lymph node metastasis [2]. Further, there is no evidence that routine lymph node dissection of the centrocervical compartment improves the survival rate [4]. However, PC is regarded as a rather aggressive disease, with recurrence rates of 40–60% [12], and this study was limited by lacking information on recurrence and whether the patients underwent repeated surgery. More thorough studies concerning the relationship between surgery and prognosis of PC are required.

In this study, 384 patients underwent parathyroidectomy, 38 patients underwent en bloc radical resection, and only 5 patients underwent debulking. Surgical debulking of tumors is a procedure whereby a surgically incurable malignant neoplasm is partially removed without curative intent in order to make subsequent therapy with drugs, radiation, or other adjunctive measures more effective and thereby improve survival. Debulking has been advocated for carcinoma of the testis and ovary, a subtype of lymphoma, sarcoma, renal cell carcinoma, adrenal and other endocrine-related tumors, neoplasms of the central nervous system, and other miscellaneous tumors [22]. However, the number of patients who underwent debulking is too small to drop accurate conclusion and further research still needs to be done to provide more actual and powerful evidence.

In addition to the limitation mentioned above, there are several unavoidable limitations in this study because the data we used was from the SEER database. First, detailed information regarding serum calcium and PTH levels of patients is unavailable, and we are not able to evaluate their relative effect on prognosis. Second, since tumor size and nodal status were not routinely collected before 1988, the missing data in this study results in the absence of several factors in the multivariate Cox regression analysis, possibly causing inadequate correction.

## 5. Conclusion

In brief, the extent of resection is related to CSS and OS in patients with PC. Factors associated with OS included black race and distant metastasis. No significant difference in prognosis was observed between patients who underwent parathyroidectomy and those who underwent en bloc resection.

## Figures and Tables

**Table 1:** Characteristics for patients with parathyroid carcinoma.

Covariate	Level	Number	%
*Age at diagnosis*	<45	131	22.1
	45–59	223	37.6
	60–69	128	21.6
	70–79	80	13.5
	>80	31	5.2

*Year of diagnosis*	1973–2004	303	51.1
	2004–2015	290	48.9

*Sex*	Female	294	49.6
	Male	299	50.4

*Race*	White	449	75.7
	Black	96	16.2
	Other	46	7.8

*Extent of diseases*	Local disease	208	35.1
	Regional disease	90	15.2
	Metastatic disease	7	1.2
	Unstaged	288	48.6

*LN status*	Negative	129	21.8
	Positive	15	2.5
	Not reported	449	75.7

*Size of tumor (cm)*	0–2.9	120	20.2
	>3	90	15.2
	Unknown	383	64.6

*Grade*	Well-differentiated	53	8.9
	Moderately differentiated	15	2.5
	Poorly differentiated	3	0.5
	Undifferentiated	2	0.3
	Unknown	520	87.7

*Radiation*	No	536	90.4
	Yes	57	9.6

*Surgery*	No	40	6.7
	Yes	553	93.3

*Extent of resection*	No definitive treatment	40	6.7
	Parathyroidectomy	384	64.8
	En bloc radical resection	38	6.4
	Debulking	5	0.8
Survival months (month)		83.00(34.00∼146.75)	
*CSS*	Alive	536	90.4
	Death	57	9.6

*OS*	Alive	385	64.9
	Death	208	35.1

**Table 2:** Univariate analyses results for clinicopathologic parameters associated with the cancer-specific survival and overall survival.

Covariate	Level	HR	CSS		*P* value	HR	OS		*P* value
			95% CI				95% CI		
*Age at diagnosis*	<45	Ref.				Ref.			
	45–59	1.423	0.552	3.668	0.465	1.903	1.171	3.092	0.009
	60–69	1.742	0.619	4.899	0.293	4.045	2.453	6.671	<0.001
	70–79	3.562	1.313	9.667	0.013	5.695	3.321	9.768	<0.001
	>80	8.818	2.651	29.338	<0.001	14.874	7.283	30.378	<0.001

*Year of diagnosis*	1973–2004	Ref.				Ref.			
	2004–2015	0.548	0.28	1.073	0.079	0.873	0.61	1.25	0.459

*Sex*	Female	Ref.				Ref.			
	Male	0.995	0.558	1.773	0.986	0.918	0.692	1.216	0.549

*Race*	White	Ref.				Ref.			
	Black	0.891	0.398	1.997	0.779	1.529	1.071	2.183	0.019
	Other	0.249	0.034	1.814	0.17	0.764	0.424	1.378	0.371

*Extent of diseases*	Local disease	Ref.				Ref.			
	Regional disease	1.433	0.419	4.899	0.566	1.398	0.761	2.566	0.28
	Metastatic disease	5.08	0.622	41.467	0.129	6.604	2.296	18.999	<0.001

*LN status*	Negative	Ref.				Ref.			
	Positive	9.06	1.828	44.898	0.007	1.735	0.721	4.172	0.219

*Size of tumor (cm)*	0–2.9	Ref.				Ref.			
	>3	5.381	1.115	25.96	0.036	1.665	0.812	3.414	0.164

*Grade*	Well-differentiated	Ref.				Ref.			
	Moderately differentiated	0.967	0.1	9.32	0.977	0.501	0.145	1.726	0.273
	Poorly differentiated	—	—	—	0.994	1.559	0.204	11.914	0.669
	Undifferentiated	—	—	—	0.995	1.273	0.165	9.798	0.817

*Radiation*	No	Ref.				Ref.			
	Yes	1.553	0.658	3.668	0.315	1.409	0.895	2.219	0.138

*Surgery*	No	Ref.				Ref.			
	Yes	0.104	0.053	0.206	<0.001	0.364	0.218	0.607	<0.001

*Extent of resection*	No definitive treatment	Ref.				Ref.			
	Parathyroidectomy	0.083	0.039	0.177	<0.001	0.299	0.175	0.511	<0.001
	En bloc radical resection	0.087	0.019	0.396	0.002	0.331	0.15	0.731	0.006
	Debulking	0.463	0.06	3.6	0.462	0.853	0.195	3.737	0.833

**Table 3:** Multivariate analyses results for clinicopathologic parameters associated with the cancer-specific survival and overall survival.

Covariate	Level	HR	CSS		*P* value	HR	OS		*P* value
			95% CI				95% CI		
*Age at diagnosis*	<45	Ref.				Ref.			
	45–59	1.646	0.172	15.725	0.665	1.057	0.424	2.635	0.906
	60–69	2.307	0.232	22.99	0.476	1.563	0.591	4.136	0.368
	70–79	5.395	0.536	54.261	0.152	1.926	0.692	5.365	0.21
	>80	—	—	—	—	2.835	0.704	11.419	0.143

*Year of diagnosis*	1973–2004	Ref.				Ref.			
	2004–2015	0.215	0.048	0.957	0.044	0.854	0.378	1.932	0.705

*Sex*	Female	Ref.				Ref.			
	Male	1.382	0.403	4.742	0.607	0.844	0.471	1.513	0.569

*Race*	White	Ref.				Ref.			
	Black	2.762	0.74	10.312	0.131	2.375	1.263	4.465	0.007
	Other	1.836	0.201	16.798	0.59	0.691	0.159	3.001	0.622

*Extent of diseases*	Local disease	Ref.				Ref.			
	Regional disease	2.234	0.568	8.786	0.25	1.505	0.769	2.947	0.233
	Metastatic disease	6.694	0.691	64.85	0.101	6.954	2.163	22.355	0.001

*Radiation*	No	Ref.				Ref.			
	Yes	1.36	0.159	11.658	0.779	1.287	0.485	3.413	0.613

*Extent of resection*	No definitive treatment	Ref.				Ref.			
	Parathyroidectomy	0.053	0.009	0.297	0.001	0.194	0.056	0.677	0.01
	En bloc radical resection	—	—	—	—	0.121	0.022	0.678	0.016
	Debulking	—	—	—	—	0.398	0.036	4.457	0.455

**Table 4:** Cancer-specific mortality and all-cause mortality of parathyroid cancer stratified by different covariates.

Covariate	Level	Number	%	1,000 person-years	95% CI
		Cancer-specific mortality		Cancer-specific mortality	
*Year of diagnosis*	1973–2004	39	12.90	8.961	6.371–12.605
	2004–2015	18	6.20	6.442	3.221–12.881

*Extent of resection*	No definitive treatment	22	55.00	45.504	21.693–95.449
	Parathyroidectomy	18	4.70	6.670	4.146–10.729
	En bloc radical resection	2	5.30	7.648	1.913–30.581
	Debulking	1	20.00	52.863	7.447–375.281

		All-cause mortality		All-cause mortality	
*Race*	White	157	35.00	36.602	31.015–43.196
	Black	39	40.60	55.869	40.652–76.781
	Other	12	26.10	28.974	16.455–51.018

*Extent of diseases*	Local disease	30	14.40	26.927	18.712–38.748
	Regional disease	16	17.80	36.893	22.241–61.196
	Metastatic disease	4	57.10	179.775	67.473–478.995

*Extent of resection*	No definitive treatment	29	72.50	78.007	44.301–137.357
	Parathyroidectomy	89	23.20	34.524	28.015–42.547
	En bloc radical resection	10	26.30	38.241	20.576–71.073
	Debulking	2	40.00	105.727	26.442–422.742

**Table 5:** Univariate analyses results for the cancer-specific survival and overall survival in patients who went through parathyroidectomy and en bloc radical resection.

Covariate	Level	HR	CSS		*P* value	HR	OS		*P* value
			95% CI				95% CI		
*Extent of resection*	Parathyroidectomy	Ref.				Ref.			
	En bloc radical resection	1.057	0.245	4.555	0.941	1.114	0.579	2.142	0.747

**Table 6:** Multivariate analyses results for clinicopathologic parameters associated with the cancer-specific survival and overall survival focusing on parathyroidectomy and en bloc radical resection in a different extent of resection.

Covariate	Level	HR	CSS		*P* value	HR	OS		*P* value
			95% CI				95% CI		
*Age at diagnosis*	<45	Ref.				Ref.			
	45–59	1.857	0.376	9.164	0.447	1.507	0.773	2.939	0.229
	60–69	4.453	0.842	23.557	0.079	3.138	1.538	6.401	0.002
	70–79	3.951	0.715	21.815	0.115	3.526	1.711	7.265	0.001
	>80	—	—	—	—	7.787	2.642	22.952	<0.001

*Year of diagnosis*	1973–2004	Ref.				Ref.			
	2004–2015	0.336	0.109	1.031	0.057	0.68	0.409	1.129	0.136

*Sex*	Female	Ref.				Ref.			
	Male	0.483	0.186	1.254	0.135	0.644	0.425	0.978	0.039

*Race*	White	Ref.				Ref.			
	Black	2.396	0.81	7.089	0.114	2.125	1.289	3.503	0.003
	Other	0.894	0.115	6.97	0.915	1.28	0.608	2.692	0.515

*Extent of diseases*	Local disease	Ref.				Ref.			
	Regional disease	1.877	0.453	7.779	0.385	1.357	0.705	2.611	0.361
	Metastatic disease	7.415	0.745	73.743	0.087	6.42	2.08	19.815	0.001

*Radiation*	No	Ref.				Ref.			
	Yes	1.034	0.23	4.646	0.965	1.74	0.948	3.193	0.074

*Extent of resection*	Parathyroidectomy	Ref.				Ref.			
	En bloc radical resection	1.183	0.261	5.367	0.827	1.225	0.62	2.421	0.56

## Data Availability

This retrospective study used data from the National Cancer Institute's Surveillance, Epidemiology, and End Results (SEER) Program. Since SEER is a publicly available database with anonymized data, no ethical review was required.

## Supplementary Materials

Supplementary Materials Supplemental Table 1: univariate analyses results for the cancer-specific survival and overall survival in patients with a different extent of resection taking debulking as reference. Supplemental Table 2: multivariate analyses results for clinicopathologic parameters associated with the cancer-specific survival and overall survival focusing on taking debulking as a reference in a different extent of resection. Supplemental Figure 1: Kaplan–Meier curves among patients without definitive treatment and patients with different types of resection for cancer-specific survival (A, C, E) and overall survival (B, D, F). Supplemental Figure 2: Kaplan–Meier curves among patients who went through parathyroidectomy and en bloc radical resection for cancer-specific survival (A) and overall survival (B). Supplemental Figure 3: Kaplan–Meier curves among patients who went through debulking and patients with other two types of resection for cancer-specific survival (A, B) and overall survival (C, D).

## References

[1] Dudney W. C., Bodenner D., Stack B. C. (2010). Parathyroid carcinoma. *Otolaryngologic Clinics of North America*.

[2] Lee P. K., Jarosek S. L., Virnig B. A., Evasovich M., Tuttle T. M. (2007). Trends in the incidence and treatment of parathyroid cancer in the United States. *Cancer*.

[3] Givi B., Shah J. P. (2010). Parathyroid carcinoma. *Clinical Oncology*.

[4] Cetani F., Pardi E., Marcocci C. (2016). Update on parathyroid carcinoma. *Journal of Endocrinological Investigation*.

[5] Shane E. (2001). Parathyroid carcinoma. *The Journal of Clinical Endocrinology & Metabolism*.

[6] Lo W. M., Good M. L., Nilubol N., Perrier N. D., Patel D. T. (2018). Tumor size and presence of metastatic disease at diagnosis are associated with disease-specific survival in parathyroid carcinoma. *Annals of Surgical Oncology*.

[7] Kebebew E. (2001). Parathyroid carcinoma. *Current Treatment Options in Oncology*.

[8] Levin K. E., Galante M., Clark O. H. (1987). Parathyroid carcinoma versus parathyroid adenoma in patients with profound hypercalcemia. *Surgery*.

[9] Owen R. P., Silver C. E., Pellitteri P. K. (2011). Parathyroid carcinoma: a review. *Head & Neck*.

[10] Campennì A., Giovinazzo S., Pignata S. A. (2017). Association of parathyroid carcinoma and thyroid disorders: a clinical review. *Endocrine*.

[11] National Cancer Institute (2017). *Parathyroid Cancer Treatment*.

[12] Ryhänen E. M., Leijon H., Metso S. (2017). A nationwide study on parathyroid carcinoma. *Acta Oncologica*.

[13] Schulte K.-M., Talat N. (2012). Diagnosis and management of parathyroid cancer. *Nature Reviews Endocrinology*.

[14] Harari A., Waring A., Fernandez-Ranvier G. (2011). Parathyroid carcinoma: a 43-year outcome and survival analysis. *The Journal of Clinical Endocrinology & Metabolism*.

[15] Schulte K. M., Talat N., Galata G. (2014). Oncologic resection achieving r0 margins improves disease-free survival in parathyroid cancer. *Annals of Surgical Oncology*.

[16] Wei C. H., Harari A. (2012). Parathyroid carcinoma: update and guidelines for management. *Current Treatment Options in Oncology*.

[17] Hara H., Igarashi A., Yano Y. (2001). Ultrasonographic features of parathyroid carcinoma. *Endocrine Journal*.

[18] Asare E. A., Sturgeon C., Winchester D. J. (2015). Parathyroid carcinoma: an update on treatment outcomes and prognostic factors from the National Cancer Data Base (NCDB). *Annals of Surgical Oncology*.

[19] Wynne A. G., Heerden J. V., Carney J. A., Fitzpatrick L. A. (1992). Parathyroid carcinoma. *Medicine*.

[20] Hundahl S. A., Fleming I. D., Fremgen A. M., Menck H. R. (1999). Two hundred eighty-six cases of parathyroid carcinoma treated in the U.S. between 1985-1995. The American College of Surgeons Commission on Cancer and the American Cancer Society. *Cancer*.

[21] Busaidy N. L., Jimenez C., Habra M. A. (2004). Parathyroid carcinoma: a 22-year experience. *Head & Neck*.

[22] Silberman A. W. (1982). Surgical debulking of tumors. *Surgery, Gynecology & Obstetrics*.

