# Supplementary material for: Surgical Disparities of Parathyroid Carcinoma: Long-Term Outcomes and Deep Excavation Based on a Large Database
Source: J Oncol. 2021 May 27;2021:8898926. doi: 10.1155/2021/8898926 (PMC8178016; doi:10.1155/2021/8898926)

Supplemental Figure 1: Kaplan Meier curves among patients without definitive treatment and patients with different types of resection for cancer-specific survival(A,C,E) and overall survival(B,D,F).

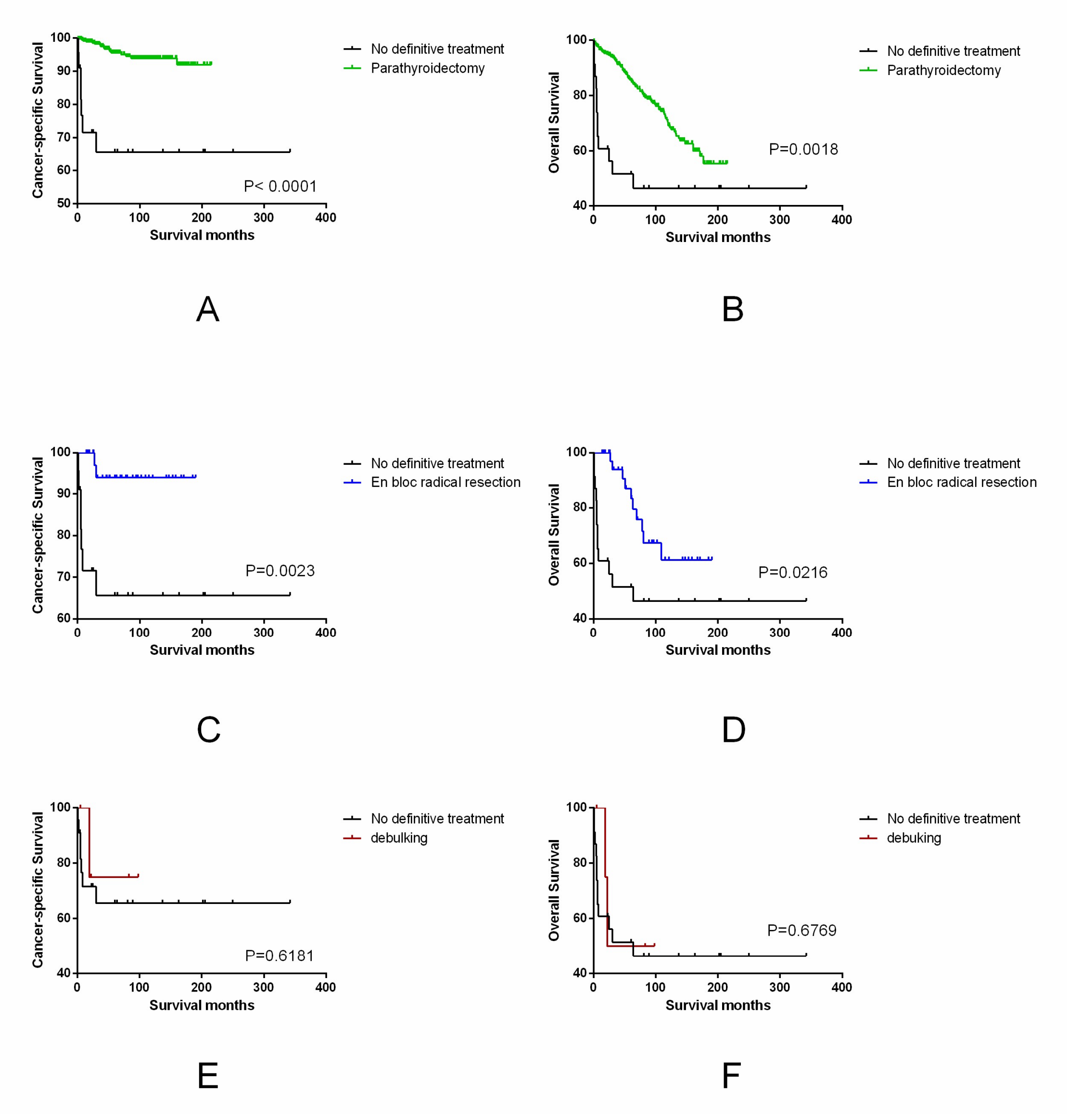

Supplemental Figure 2: Kaplan Meier curves among patients went through parathyroidectomy and en bloc radical resection for cancer-specific survival(A) and overall survival(B).

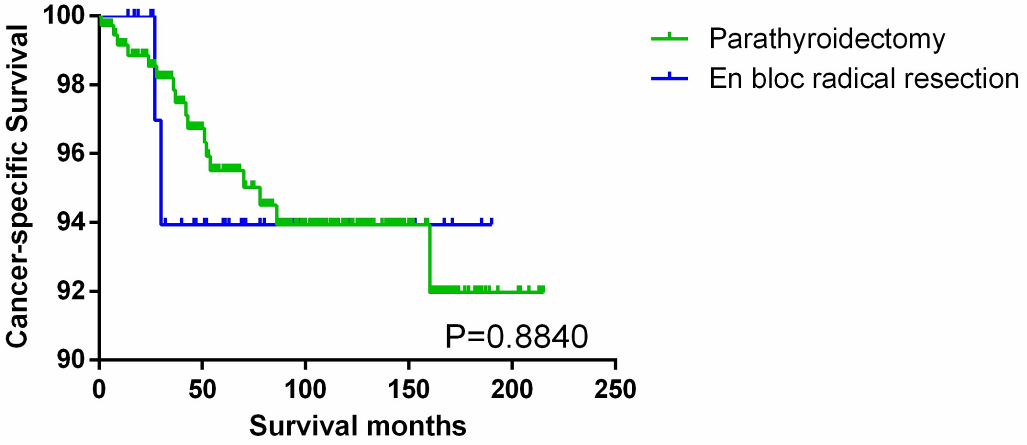


A

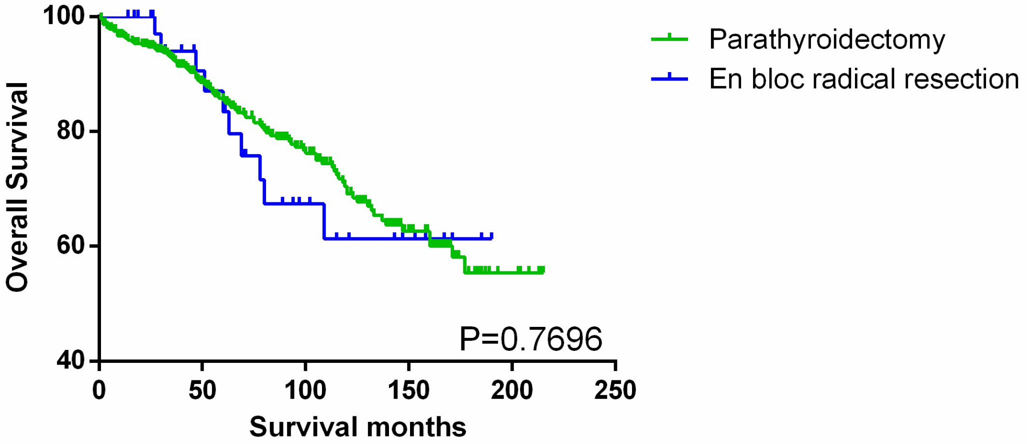


B

Supplemental Figure 3: Kaplan Meier curves among patients went through debulking and patients with other two types of resection for cancer-specific survival(A,B) and overall survival(C,D).

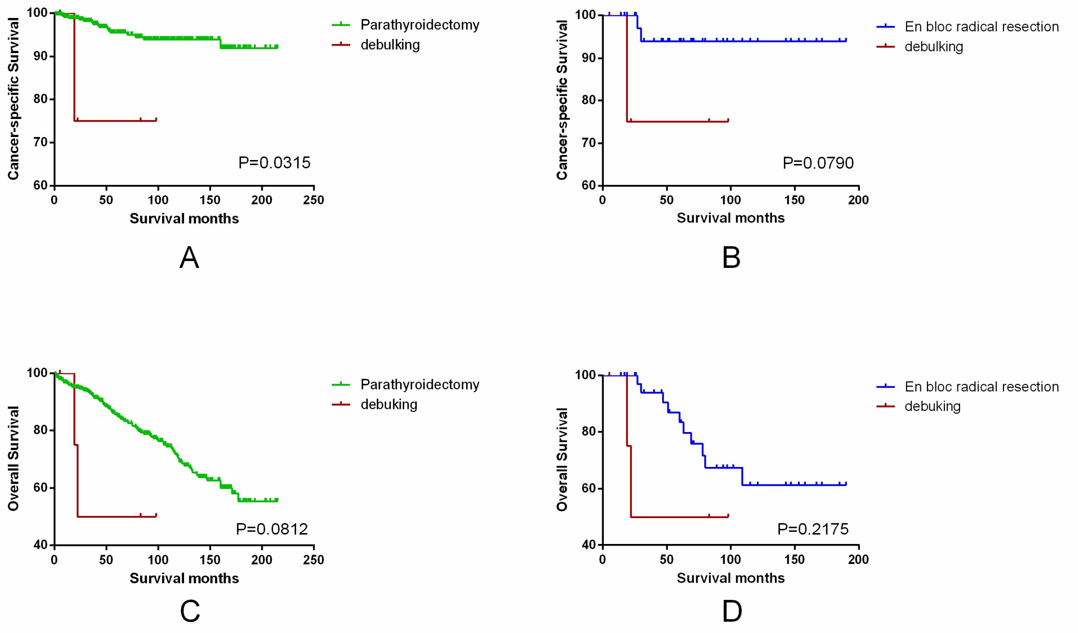

Supplement: Supplementary Materials — Supplemental Table 1: univariate analyses results for the cancer-specific survival and overall survival in patients with a different extent of resection taking debulking as reference. Supplemental Table 2: multivariate analyses results for clinicopathologic parameters associated with the cancer-specific survival and overall survival focusing on taking debulking as a reference in a different extent of resection. Supplemental Figure 1: Kaplan–Meier curves among patients without definitive treatment and patients with different types of resection for cancer-specific survival (A, C, E) and overall survival (B, D, F). Supplemental Figure 2: Kaplan–Meier curves among patients who went through parathyroidectomy and en bloc radical resection for cancer-specific survival (A) and overall survival (B). Supplemental Figure 3: Kaplan–Meier curves among patients who went through debulking and patients with other two types of resection for cancer-specific survival (A, B) and overall survival (C, D). [file 8898926.f1.zip › 8898926.f1/Supplemental Figures (1).pdf]
