# Supplementary material for: Surgical Disparities of Parathyroid Carcinoma: Long-Term Outcomes and Deep Excavation Based on a Large Database
Source: J Oncol. 2021 May 27;2021:8898926. doi: 10.1155/2021/8898926 (PMC8178016; doi:10.1155/2021/8898926)
Supplement: Supplementary Materials — Supplemental Table 1: univariate analyses results for the cancer-specific survival and overall survival in patients with a different extent of resection taking debulking as reference. Supplemental Table 2: multivariate analyses results for clinicopathologic parameters associated with the cancer-specific survival and overall survival focusing on taking debulking as a reference in a different extent of resection. Supplemental Figure 1: Kaplan–Meier curves among patients without definitive treatment and patients with different types of resection for cancer-specific survival (A, C, E) and overall survival (B, D, F). Supplemental Figure 2: Kaplan–Meier curves among patients who went through parathyroidectomy and en bloc radical resection for cancer-specific survival (A) and overall survival (B). Supplemental Figure 3: Kaplan–Meier curves among patients who went through debulking and patients with other two types of resection for cancer-specific survival (A, B) and overall survival (C, D). [file 8898926.f1.zip › 8898926.f1/Supplemental Tables.docx]

Supplemental Table 1: Univariate analyses results for the cancer-specific survival and overall survival in patients with different extent of resection taking debulking as reference.

|  |  |  |  |  |  |  |  |  |  |
| --- | --- | --- | --- | --- | --- | --- | --- | --- | --- |
| **Covariate** | **level** | **CSS** | | | | **OS** | | | |
|  |  | **HR** | **95 % CI** | | **p value** | **HR** | **95 % CI** | | **p value** |
| Extent of resection | debulking | ref |  |  |  | ref |  |  |  |
|  | En bloc radical resection | 0.156 | 0.021 | 1.179 | 0.072 | 0.317 | 0.078 | 1.294 | 0.109 |
|  | Parathyroidectomy | 0.165 | 0.015 | 1.825 | 0.142 | 0.352 | 0.077 | 1.615 | 0.179 |

Supplemental Table 2: Multivariate analyses results for clinicopathologic parameters associated with the cancer-specific survival and overall survival focusing on taking debulking as reference in different extent of resection.

|  |  |  |  |  |  |  |  |  |  |
| --- | --- | --- | --- | --- | --- | --- | --- | --- | --- |
| **Covariate** | **level** | **CSS** | | | | **OS** | | | |
|  |  | **HR** | **95 % CI** | | **p value** | **HR** | **95 % CI** | | **p value** |
| Age at diagnosis | <45 | ref |  |  |  | ref |  |  |  |
|  | 45–59 | 1.902 | 0.385 | 9.385 | 0.43 | 1.517 | 0.778 | 2.958 | 0.221 |
|  | 60–69 | 4.575 | 0.864 | 24.237 | 0.074 | 3.145 | 1.542 | 6.414 | 0.002 |
|  | 70–79 | 3.836 | 0.695 | 21.155 | 0.123 | 3.476 | 1.688 | 7.158 | 0.001 |
|  | >80 | - | - | - | - | 7.7 | 2.615 | 22.678 | <0.001 |
| Year of diagnosis | 1973-2004 | ref |  |  |  | ref |  |  |  |
|  | 2004-2015 | 0.298 | 0.096 | 0.925 | 0.036 | 0.656 | 0.395 | 1.088 | 0.103 |
| Sex | Female | ref |  |  |  | ref |  |  |  |
|  | Male | 0.461 | 0.181 | 1.176 | 0.105 | 0.643 | 0.427 | 0.97 | 0.035 |
| Race | White | ref |  |  |  | ref |  |  |  |
|  | Black | 2.519 | 0.854 | 7.436 | 0.094 | 2.131 | 1.294 | 3.508 | 0.003 |
|  | Other | 0.891 | 0.115 | 6.933 | 0.912 | 1.265 | 0.602 | 2.657 | 0.534 |
| Extent of diseases | Local disease | ref |  |  |  | ref |  |  |  |
|  | Regional disease | 1.437 | 0.316 | 6.536 | 0.639 | 1.291 | 0.667 | 2.499 | 0.449 |
|  | Metastatic disease | 7.66 | 0.763 | 76.852 | 0.084 | 6.332 | 2.055 | 19.513 | 0.001 |
| Radiation | NO | ref |  |  |  | ref |  |  |  |
|  | Yes | 0.935 | 0.21 | 4.157 | 0.93 | 1.618 | 0.883 | 2.967 | 0.119 |
| Extent of resection | debulking | ref |  |  |  | ref |  |  |  |
|  | En bloc radical resection | 0.076 | 0.008 | 0.749 | 0.027 | 0.21 | 0.047 | 0.95 | 0.043 |
|  | Parathyroidectomy | 0.092 | 0.007 | 1.268 | 0.075 | 0.26 | 0.052 | 1.296 | 0.1 |
